# Meta-analysis of intravoxel incoherent motion magnetic resonance imaging in differentiating focal lesions of the liver

**DOI:** 10.1097/MD.0000000000012071

**Published:** 2018-08-24

**Authors:** Hongzhen Wu, Yingying Liang, Xinqing Jiang, Xinhua Wei, Yu Liu, Weifeng Liu, Yuan Guo, Wenjie Tang

**Affiliations:** a Department of Radiology, Guangzhou First People's Hospital, School of Medicine, South China University of Technology, Guangzhou, Guangdong; b Guangzhou First People's Hospital, Guangzhou Medical University, Guangzhou, China.

**Keywords:** diffusion-weighted magnetic resonance imaging, liver neoplasms, magnetic resonance imaging, meta-analysis, perfusion-weighted magnetic resonance imaging

## Abstract

**Introduction:**

Accurate detection and characterization of focal liver lesions, including differentiation between malignant and benign lesions, are particularly important. The objective of this meta-analysis was to evaluate the parameters of intravoxel incoherent motion (IVIM), including apparent diffusion coefficient (ADC), pure molecular diffusion coefficient (*D*), perfusion-related diffusion coefficient (*D*∗), and perfusion fraction (*f*) in differentiating focal liver lesions.

**Methods:**

IVIM method employed for focal liver lesion and the quality assessment of diagnostic studies were evaluated. Standardized mean differences and 95% confidence intervals were calculated. The heterogeneity was quantified with the *I*^2^ statistic.

**Results:**

The difference between groups was analyzed according to the *I*^2^ values from 6 different studies using fixed effects or random effects models. Significant differences in ADC (*P* < .001) and *D* (*P* < .001) were observed between benign and malignant lesions. Moreover, significant differences in ADC (*P* < .001), *D* (*P* < .001), and *f* (*P* = .01) were found between hemangioma and hepatocellular carcinoma (HCC). In addition, no significant difference was observed between the metastases and HCC.

**Conclusions:**

*D* and ADC values were useful for the differentiation between benignity and malignancy; higher values of ADC, *D*, and *f* were observed in hemangioma compared to HCC. Nevertheless, IVIM did not result as the optimal approach for differentiation between the metastases and HCC.

## 1 Introduction

Noninvasive and real-time imaging methods provide a useful tool for investigating the pathological information on focal liver lesions.^[1–5]^ The accurate detection and characterization of those lesions, including accurate differentiation between malignant and benign lesions, are of particular importance.

Qualitative analysis of diffusion-weighted magnetic resonance (MR) images has become increasingly popular for the evaluation of various liver diseases.^[6]^ Intravoxel incoherent motion (IVIM) imaging, a method based on diffusion-weighted imaging (DWI) with multiple *b* values representing the degree of diffusion weighting,^[7]^ allows for the separate analysis of 2 components of random water motion in biological tissue, that is, pure molecular diffusion and microcirculation (or perfusion), with the parameters of pure molecular diffusion coefficient (*D*), perfusion fraction (*f*), and perfusion-related diffusion coefficient (*D*∗).^[8–10]^

IVIM is becoming ever more popular in clinical research as it provides the additional perfusion information without requiring extensive changes in the MR acquisition protocols.^[8,11–14]^ Moreover, IVIM imaging has recently been used for liver imaging,^[15]^ where it has shown to be useful for evaluation of liver fibrosis, nonalcoholic fatty liver disease, and focal liver lesions.^[16–19]^ Furthermore, besides being a good approach for cancerous tumors, IVIM is useful for estimating the diffusion and perfusion of tumor tissue.^[20,21]^ Increased cell density and increased angiogenesis are important pathological processes, accompanied by many types of malignant tumors.^[9,22]^ Nevertheless, different research studies have shown very contradictory data regarding the usage of IVIM for focal liver lesions diagnosis; calling for further investigations into the matter.^[22–26]^

The aim of this study was to review published data related to IVIM parameters including apparent diffusion coefficient (ADC), *D*, *D*∗, and *f* values, and to evaluate the differences in focal liver lesions among different patients.

## 2 Methods

All analyses were based on previous published studies; thus, no ethical approval and patient consent are required.

### 2.1 Data sources and keywords

To identify relevant published studies that evaluated the diagnostic value of focal liver lesions, PubMed, Cochrane Library, MEDLINE, Web of Science, EMBASE, and CNKI databases (last updated search: November 1, 2016; data included Chinese and English language articles) were comprehensively explored by 4 experienced radiologists (HW, YL, YG, and WT). The following search terms were used: “liver and intravoxel incoherent motion MR imaging,” “liver and IVIM,” “hepatic or hepatology and intravoxel incoherent motion MR imaging,” “hepatic or hepatology and IVIM,” “liver lesions and intravoxel incoherent motion MR imaging,” “liver lesions and IVIM,” “hepatic lesions and intravoxel incoherent motion MR imaging,” and “hepatic lesions and IVIM.”, “liver or hepatic lesions and DWI or diffusion-weighted imaging.” In addition, bibliographies from prominent studies were searched manually to identify additional relevant studies.

### 2.2 Quality assessment

The quality assessment of diagnostic studies (QUADAS) was used by 2 independent reviewers (attending radiologists for body imaging with 10 and 17 years of clinical experience, respectively) to assess the quality of each study to be included in this meta-analysis.^[27–29]^ Each item was assigned with “yes,” “no,” or “unclear” (if there was insufficient information to make an accurate judgment) based on QUADAS-2 score. Disagreements were resolved by consensus. All assessment results were imported into RevMan (version 5.2) software.

### 2.3 Assessment of reporting biases

Since none of the meta-analysis included 10 or more studies, we did not assess publication bias using a funnel plot.^[30,31]^ We performed a comprehensive search strategy to reduce the potential for publication bias.

### 2.4 Eligibility criteria

Two reviewers who were blinded to the journal, author, institution, and date of publication, independently screened the titles and abstracts and assessed the full text to identify potentially eligible articles; disagreements were resolved by consensus. Studies were included in this analysis if IVIM MR imaging were obtained using either a 1.5 or 3.0-T MR scanner; the diagnostic criteria of the benign and malignant liver lesions were clearly stated; IVIM analysis methods were reported; ADC, *D*, *D*∗, and *f* (%) mean value of hepatic lesions for benign and malignant were summarized. The exclusion criteria included not original research (reviews, editorials, and nonresearch letters); the incomprehensive data; and no summary of benign and malignant or no classification.

### 2.5 Data extraction

Two reviewers separately collected information from eligible studies. The following data were collected: first author, publication year, study design, ethnicity, number of participants, age, sex, number of lesions, and mean value of the ADC, *D*, *D*∗, and *f* (%). Authors of abstracts and studies with insufficient data were contacted to collect additional information regarding their studies.

### 2.6 Statistical analysis

For the IVIM parameters [ADC, *D*, *D*∗, and *f* (%)] mean and standard deviation (SD) were extracted or derived using the reported data. To analyze the differences between groups, 2 different approaches were used: fixed-effect and random-effect models. All meta-analyses were performed using a fixed-effect or random-effect model according to the *I*^2^ values. Heterogeneity was quantified with the *I*^2^, which describes the proportion of the total variation in study estimates caused by heterogeneity.^[32,33]^ If the *I*^2^ value was <50%, the heterogeneity was considered acceptable and fixed-effect model was used; and if the *I*^2^ value was >50%, it implied the existence of heterogeneity and random-effect models was used.^[34]^ For continuous variables, standardized mean difference (SMD) and 95% confidence intervals (CIs) were calculated. Statistical analyses were conducted using Review Manager (version 5, The Cochrane Collaboration). For all tests, *P* values <0.05 indicated statistically significant differences.

In this study, 3 main outcome measurements were calculated. Primarily, we assessed the difference of the mean value of IVIM parameters between benign and malignant focal lesions. Then, we focused on the IVIM parameters mean differences between hemangioma and hepatocellular carcinoma (HCC). Finally, we examined performance of IVIM parameters in distinguishing metastases from HCC.

## 3 Results

### 3.1 Study selection and data extraction

Titles and abstracts from retrieved references were screened to identify potentially eligible articles for inclusion in the review, whereas potentially relevant full text articles were analyzed based on the inclusion criteria. The initial database search identified 586 relevant articles that were published after November 1, 2016. Consequently, 6 articles were selected for data extraction (Fig. 1 A). Details of QUADAS are shown in Figure 1 B and C.

**Figure 1:**
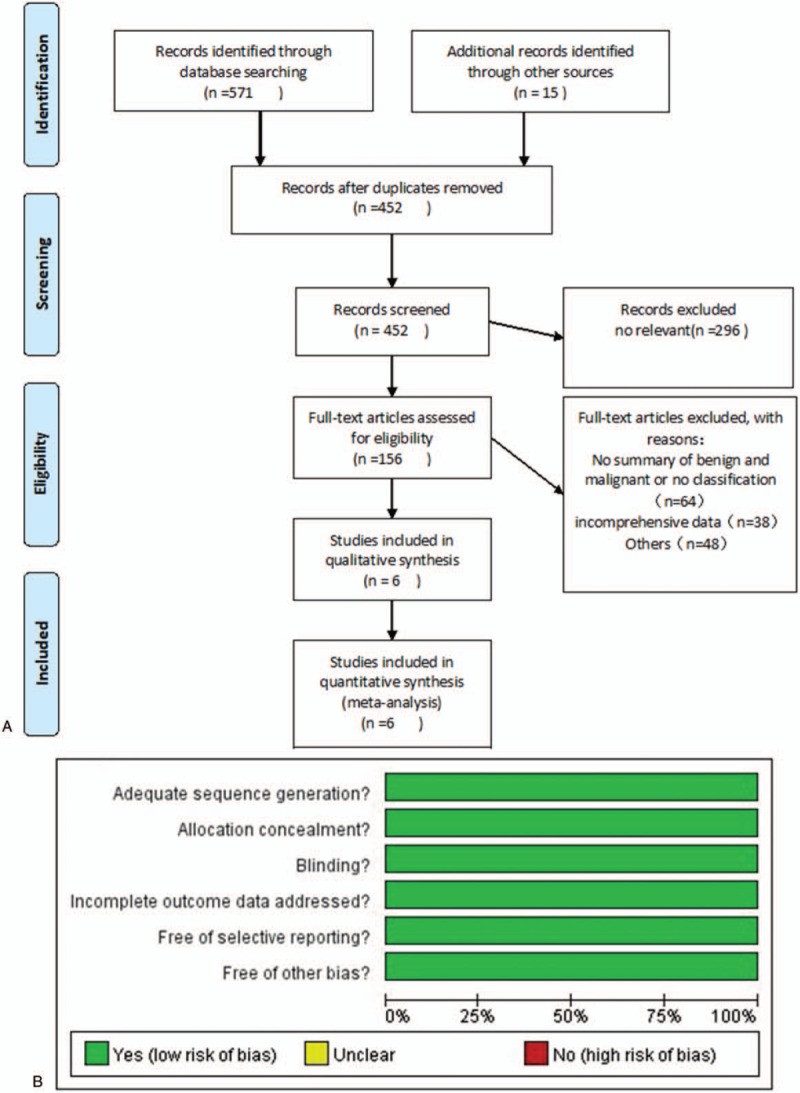
A, Study selection process. The flowchart summarizes the selection of studies including numbers and reasons of exclusion. B, Risk of bias graph: review authors’ judgments about each risk of bias item presented as percentages across all included studies. C, Risk of bias summary: review authors’ judgments about each risk of bias item for each included study.

**Figure 1 (Continued):**
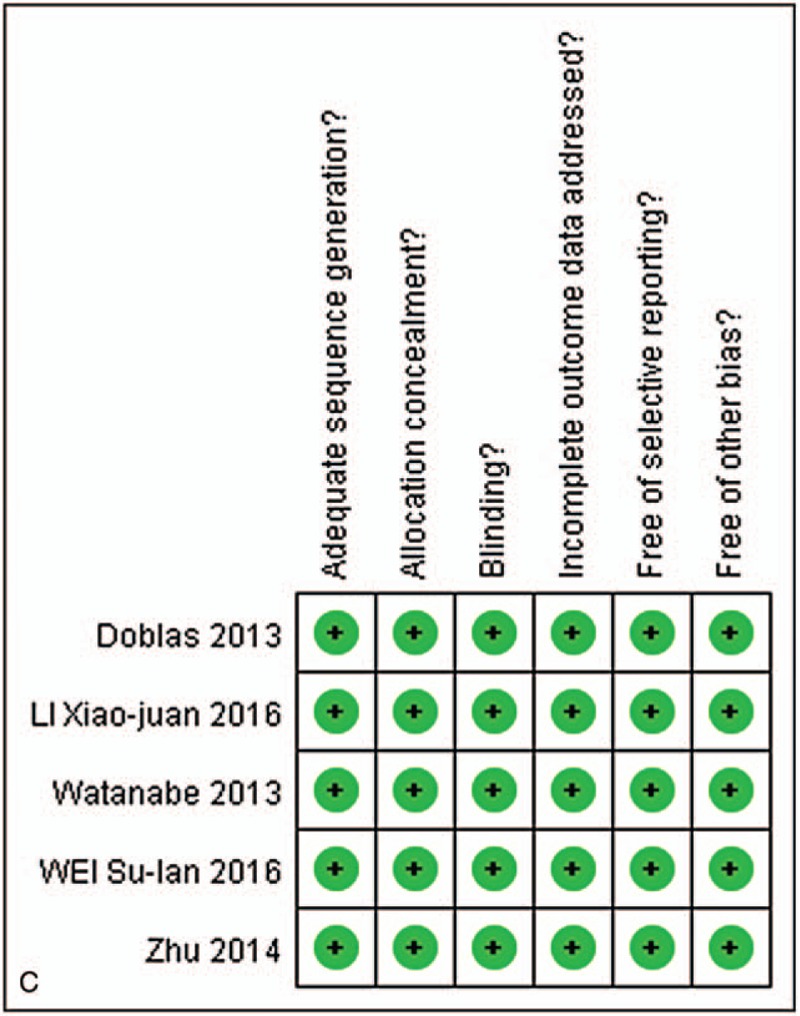
A, Study selection process. The flowchart summarizes the selection of studies including numbers and reasons of exclusion. B, Risk of bias graph: review authors’ judgments about each risk of bias item presented as percentages across all included studies. C, Risk of bias summary: review authors’ judgments about each risk of bias item for each included study.

### 3.2 Description of the studies

A meta-analysis database was established according to the extracted information from each selected article. Study subjects, study baseline characteristics, and methodological qualities are shown in Table 1.

**Table 1:**
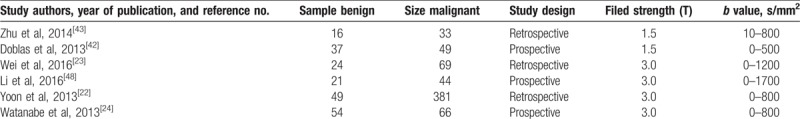
Characteristics of included studies.

This meta-analysis was performed on the per-lesion basis. Six articles included a total of 484 patients with 582 liver lesions, including 381 malignant and 201 benign lesions. From 381 malignancies, 257 lesions were HCCs, 102 were metastases and 22 were cholangiocellular carcinoma. The benign lesions included 100 hemangiomas, 44 cysts, 37 focal nodular hyperplasia, 14 adenomas, 5 abscesses, and 1 angiomyolipoma. All liver lesions were confirmed by pathology and/or overall analysis combined with medical history, clinical symptoms, and various imaging data.

### 3.3 Performance of IVIM parameters in distinguishing benign from malignant lesions

**Figure 2:**
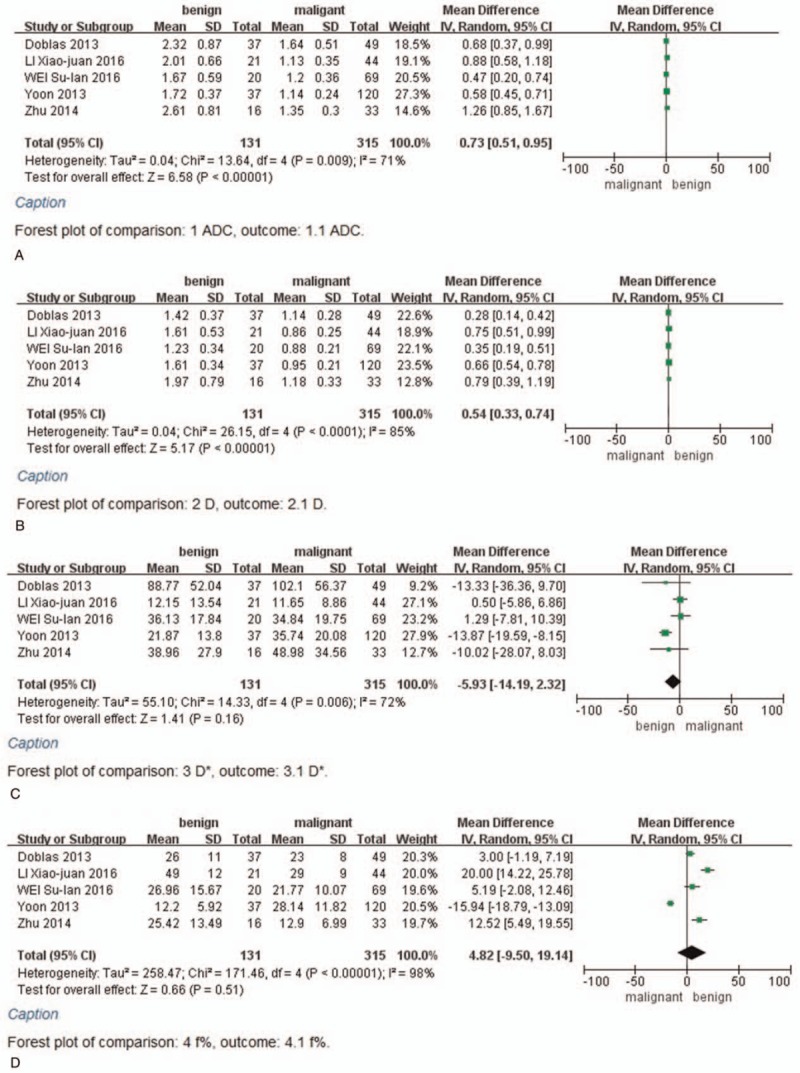
A, Forest plot showing results of the mean apparent diffusion coefficient (ADC) value between benign and malignant focal liver lesions [mean ADC ± standard deviation (SD) × 10^–3^ mm^2^/s]. B, Forest plot showing results of the mean *D* value between benign and malignant focal liver lesions (mean *D* ± SD × 10^–3^ mm^2^/s). C, Forest plot showing results of the mean *D*^∗^ value between benign and malignant focal liver lesions (mean *D*^∗^ ± SD × 10^–3^ mm^2^/s). D, forest plot showing results of the mean *f* value between benign and malignant focal liver lesions [mean *f*(%) ± SD].

Five of 6 studies evaluated the performance of parameters comparing benign with malignant lesions. The random-effect model (*I*^2^ > 50%) and SMD were used to perform the meta-analysis. Briefly, the ADC, *D*, *D*∗, and *f* (%) results indicated that the weight of included studies ranged from 14.6% to 27.3%, 12.8% to 23.5%, 9.2% to 27.9%, and 19.6% to 20.5%. The weight derived from SD, which indicates the weight of each study in the combined effect volume can be used to evaluate the quality of references.^[35]^ The values of ADC, *D*, *D*∗, and *f* (%) in benign compared with malignant lesions were 7.3 × 10^–4^ mm^2^/s [95% CI = (5.1 – 9.5) × 10^–4^ mm^2^/s; test for heterogeneity = 13.64, *P* = .009, *I*^2^ = 71%, test for overall effect: *Z* = 6.58, *P* < .001] (Fig. 2A); 5.4 × 10^–4^ mm^2^/s [95% CI = (3.3 – 7.4) × 10^–4^ mm^2^/s; test for heterogeneity = 26.15, *P* < .001, *I*^2^ = 85%; test for overall effect: *Z* = 5.17, *P* < .001] (Fig. 2B); −5.93 × 10^–3^ mm^2^/s [95% CI = (−14.19–2.32) × 10^–3^ mm^2^/s; test for heterogeneity = 14.33, *P* = .006, *I*^2^ = 72%; test for overall effect: *Z* = 1.41, *P* = .16) (Fig. 2C); 4.82(95% CI = −9.50–19.14; test for heterogeneity = 171.46, *P* < .001, *I*^2^ = 98%; test for overall effect: *Z* = 0.66, *P* = .51) (Fig. 2D). Furthermore, ADC and *D* values were significantly higher in benign lesions, while there was no significant difference in the *D*∗ and *f* values between the benign and malignant lesions.

### 3.4 Performance of IVIM parameters in distinguishing hemangioma from hepatocellular carcinoma

**Figure 3:**
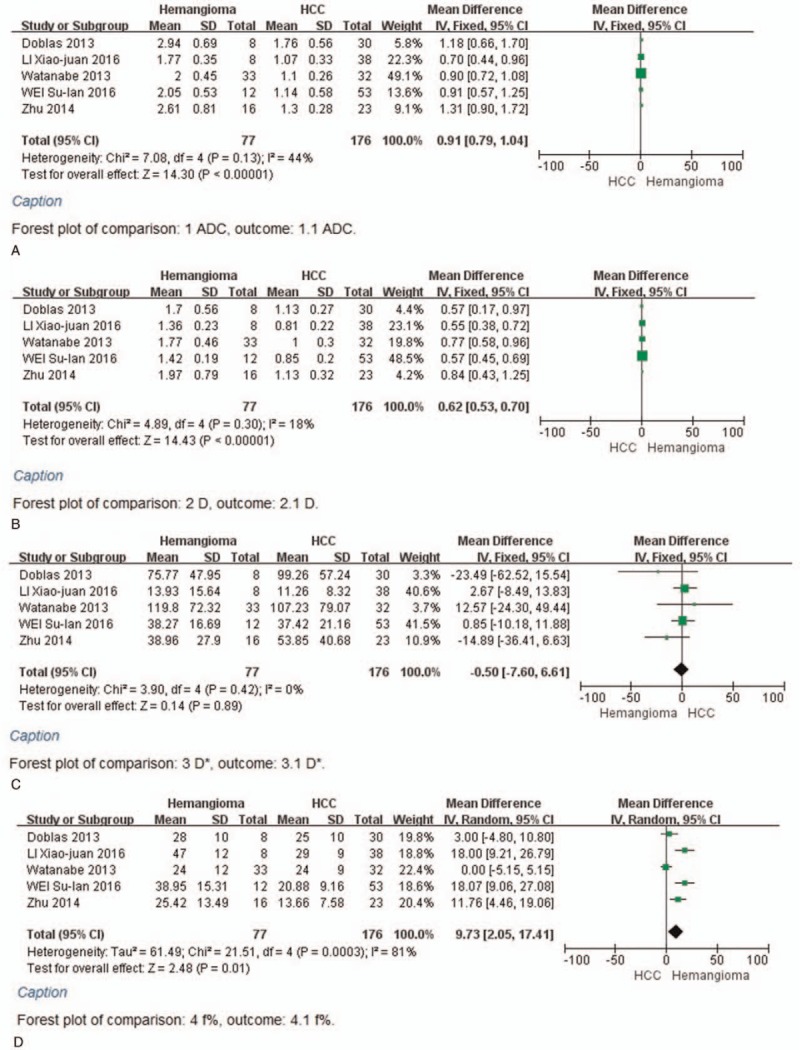
A, Forest plot showing results of the mean apparent diffusion coefficient (ADC) value between hemangioma and hepatocellular carcinoma [mean ADC ±  standard deviation (SD)×10^–3^ mm^2^/s]. B, Forest plot showing results of the mean *D* value between hemangioma and hepatocellular carcinoma (mean *D* ± SD × 10^–3^ mm^2^/s). C, Forest plot showing results of the mean *D*^∗^ value between hemangioma and hepatocellular carcinoma (mean *D*^∗^ ± SD × 10^–3^ mm^2^/s). D, Forest plot showing results of the mean *f* value between hemangioma and hepatocellular carcinoma [mean *f* (%) ± SD].

Five of 6 studies evaluated the performance of parameters in hemangioma compared with HCC. The fixed-effect mode for ADC, *D*, and *D*∗ values (*I*^2^ < 50%); the random-effect model (*I*^2^ > 50%) for *f* values; and SMD were used to perform the meta-analysis. The results of ADC, *D*, *D*∗, and *f* (%) indicated that the weight of included studies ranged from 5.8% to 49.1%, 4.2% to 48.5%, 3.3% to 41.5%, and 18.6% to 22.4%. The values of ADC, *D*, *D*∗, and *f* (%) in hemangioma compared with HCC were 9.1 × 10^–4^ mm^2^/s [95% CI = (7.9 – 10.4) × 10^–4^ mm^2^/s; test for heterogeneity = 7.08, *P* = .13, *I*^2^ = 44%, test for overall effect: *Z* = 14.30, *P* < .001] (Fig. 3A); 6.2 × 10^–4^ mm^2^/s [95% CI = (5.3 – 7.0) × 10^–4^ mm^2^/s; test for heterogeneity = 4.89, *P* = .30, *I*^2^ = 18%; test for overall effect: *Z* = 14.43, *P* < .001] (Fig. 3B); −5 × 10^–4^ mm^2^/s [95% CI = (−7.6 – 6.61) × 10^–3^ mm^2^/s; test for heterogeneity = 3.9, *P* = .42, *I*^2^ = 0%; test for overall effect: *Z* = 0.14, *P* = .89] (Fig. 3C); 9.73 (95% CI = 2.05–17.41; test for heterogeneity = 21.51, *P* = .003, *I*^2^ = 81%; test for overall effect: *Z* = 2.48, *P* = .01) (Fig. 3D). In addition, ADC, *D*, and *f* values were significantly higher in hemangioma compared to HCC, whereas there was no significant difference in the *D*∗ values between the hemangioma and HCCs.

### 3.5 Performance of IVIM parameters in distinguishing metastases from hepatocellular carcinoma

**Figure 4:**
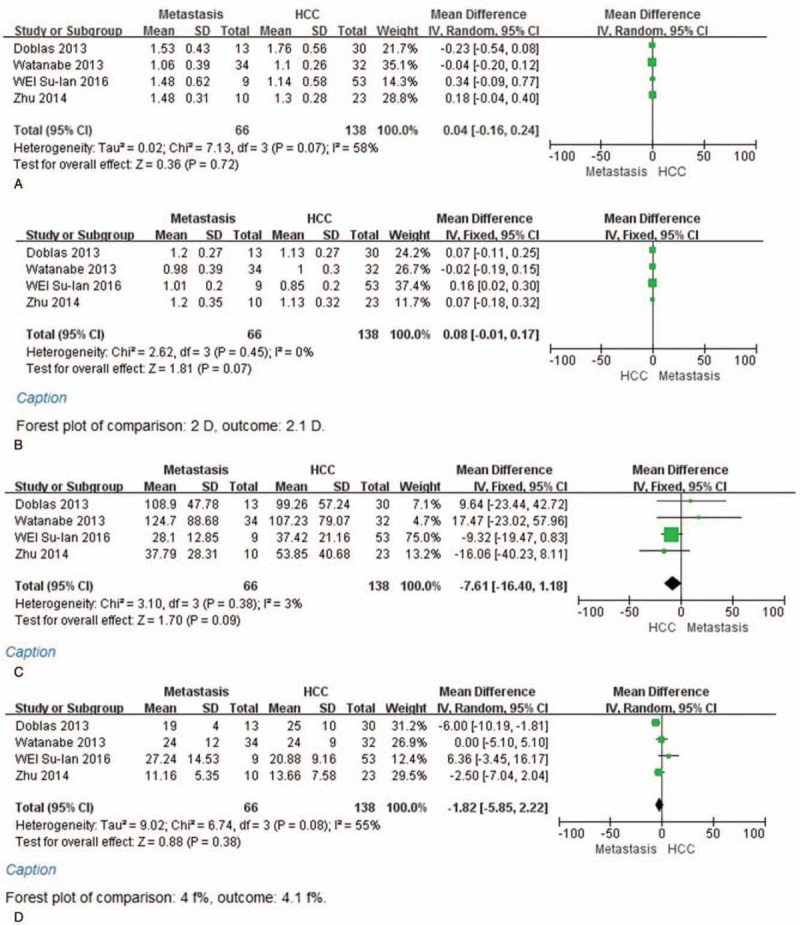
A, Forest plot showing results of the mean apparent diffusion coefficient (ADC) value between metastases and hepatocellular carcinoma [mean ADC ± standard deviation (SD) × 10^–3^ mm^2^/s]. B, Forest plot showing results of the mean *D* value between metastases and hepatocellular carcinoma (mean *D* ± SD × 10^–3^ mm^2^/s). C, Forest plot showing results of the mean *D*^∗^ value between metastases and hepatocellular carcinoma (mean *D*^∗^ ± SD × 10^–3^ mm^2^/s). D, Forest plot showing results of the mean *f* value between metastases and hepatocellular carcinoma [mean *f* (%) ± SD].

Four of 6 studies evaluated the performance of parameters in metastases compared with HCC. The fixed-effect mode for *D* and *D*∗ values because of *I*^2^ < 50%, the random-effect model (*I*^2^  > 50%) for ADC and *f* values and SMD were used to perform the meta-analysis. The results of ADC, *D*, *D*∗, and *f* (%) indicated that the weight of included studies ranged from 14.3% to 28.8%, 11.7% to 37.4%, 4.7% to 75.0%, and 12.4% to 31.4%. The values of ADC, *D*, *D*∗, and *f* (%) in metastases compared with HCC were 4 × 10^–5^ mm^2^/s [95% CI = (−1.6–2.4) × 10^–4^ mm^2^/s; test for heterogeneity = 7.13, *P* = .07, *I*^2^ = 58%, test for overall effect: *Z* = 0.36, *P* = .72] (Fig. 4A); 8 × 10^–5^ mm^2^/s [95% CI = (−1–17) × 10^–5^ mm^2^/s; test for heterogeneity = 2.62, *P* = .45, *I*^2^ = 0%; test for overall effect: *Z* = 1.81, *P* = .07] (Fig. 4B); −7.61 × 10^–3^ mm^2^/s [95% CI = (−16.4–1.18) × 10^–3^ mm^2^/s; test for heterogeneity = 3.1, *P* = .38, *I*^2^ = 3%; test for overall effect: *Z* = 1.70, *P* = .09] (Fig. 4C); −1.82 (95% CI = −5.85–2.22; test for heterogeneity = 6.74, *P* = .08, *I*^2^ = 55%; test for overall effect: *Z* = 0.88, *P* = .38) (Fig. 4D). Furthermore, there were no significant differences in the ADC, *D*, *D*∗, and *f* values between the metastases and HCC.

## 4 Discussion

IVIM imaging, or DWI with a range of low (i.e., <50 s/mm^2^) and high (i.e., >200 s/mm^2^) *b*-values, was proposed to separately measure diffusion and perfusion-related diffusion.^[25,36]^ IVIM makes it possible to obtain the true diffusion coefficient (*D*) reflecting cell density and the perfusion fraction (*f*) reflecting the microcirculation of tumors.^[37]^

The IVIM parameters *D*∗ and *f* describe the microcirculation effect. The *D*∗ value depends on the mean blood velocity and the length of the microvascular segment, and the diffusion coefficient of the blood, whereas *f* represents the fraction of the signal originating from perfusion and is expected to reflect the fractional blood volume of capillaries.^[38,39]^

Our results showed that *D* and ADC values were helpful for the differentiation between benignity and malignancy according to IVIM MR images, which suggested that true and apparent molecular diffusions may be more informative than pseudodiffusion (*D*∗) or perfusion fraction (*f*) in the characterization of liver lesions. The cellular density of malignancy was higher than benignity, whereas the ADC and *D* values were lower. Mungai et al^[40]^ have reported that ADC is useful in the classification of more than half of noncystic focal liver lesions.

The reason why there was no significant difference in *D*∗ and *f* values between liver lesions remains unclear, nevertheless blood volume (*f*), blood flow (*D*∗), or secretion could have different effects on perfusion properties in different lesion types.^[24,41]^ For example, metastases and cholangiocarcinomas with low blood supply may be highly cellular and lowly perfused compared with benign tumors.^[42]^

Secondly, ADC, *D,* and *f* values were significantly higher in hemangioma compared to HCC, whereas there was no significant difference in the *D*∗ values between the hemangioma and HCC. Because *D*∗ depends on the mean blood velocity and length of microvessel segments (and on the diffusion coefficient of blood),^[38,39]^ and given there are 3 types of hemangiomas: sufficient blood supply, lower blood supply, and lack of blood supply, the value of *D*∗ might fluctuate over a large range.^[43]^ When the blood supplies of high-flow and middle-flow hemangiomas come from the hepatic artery, they are similar to HCC's blood supply, and they might actually explain why there was no difference in *D*∗ value between the hemangioma and HCC. Nevertheless, the cellular density and blood volume were different between the hemangioma and HCC, so that the ADC, *D*, and *f* values of hemangioma were higher compared to HCC.

Finally, malignant liver tumors can be classified into primary cancers and secondary (metastatic) tumors.^[44]^ In this study, the metaregression analysis indicated there were no significant differences in the ADC, *D*, *D*∗, and *f* values between the metastases and HCCs. The metastases arise from several primary neoplasms such as gastrointestinal, lung, breast, and genitourinary,^[45]^ and may cause a number of variations in the cell density and microcirculation. IVIM parameters are somewhat correlated with histological grade of HCC because of the differences in the cell density and microscopic circulation,^[9,41,46,47]^ nevertheless the studies included in this metaregression analysis did not report on different metastases cell types and HCC histological grades. Consequently, this might explain why there was no difference between the metastases and HCC in IVIM parameters, and it should be addressed by further research.

Since there were <5 studies included in the present data analysis, we did not make the funnel plot for publication bias, because previous studies have reported that funnel plot is not significant with <10 studies.^[30]^ We tried to collect more studies to reduce publication bias. The weight derived from SD indicates the weight of each study in the combined effect volume, and can be used to evaluate the quality of references.^[35]^ In our study, the weight was determined according to the number of cases in each study; the higher the weight, the larger the sample size was.

Our meta-analysis had several limitations. First, one of the relevant studies^[48]^ failed to include the cysts into the benign focal lesions of liver, which might have led to some biased results. Second, the IVIM model is less stable than the monoexponential diffusion model, and it requires the fitting of more variables.^[11,49]^ Free-breathing or respiratory-triggered, multi-*b* values and cardiac motion artifacts may affect the measurement repeatability of IVIM parameters.^[6,50]^ In the studies we used, the MR scanning parameters were not consistent; 6 studies all together^[22,24,42,23,43,48]^ did not have unified *b* values and the field strength, which in turn had impact on the results of the meta-analysis. Since IVIM is somewhat a new technology, there are relatively fewer published articles; therefore, this article can serve as a preliminary study. We will continue to follow and collect relevant studies for future analyses.

In conclusion, *D* and ADC values were helpful for the differentiation between benignity and malignancy on IVIM MR imaging, and thus indicating that true and apparent molecular diffusions may be more informative than pseudodiffusion (*D*∗) or perfusion fraction (*f*) in the characterization of liver lesions. Secondly, the ADC, *D*, and *f* values of hemangioma were higher compared to HCC, whereas *D*∗ value showed no difference. This might be due to the types of various blood supplies of hemangioma. However, because of different metastatic cell types and HCC histological grades, IVIM was not very helpful for differentiating the metastases and HCC in the present study, and thus calling for further verifications in the future.

## Acknowledgments

The authors thank Lijun Ouyang for proofreading the manuscript.

## Author contributions

**Data curation:** Hongzhen Wu, Xinqing Jiang.

**Formal analysis:** Hongzhen Wu.

**Investigation:** Hongzhen Wu, Yingying Liang, Xinqing Jiang, Xinhua Wei, Weifeng Liu, Yuan Guo, Wenjie Tang.

**Methodology:** Hongzhen Wu, Yingying Liang, Xinqing Jiang, Xinhua Wei, Weifeng Liu, Yuan Guo, Wenjie Tang.

**Resources:** Hongzhen Wu.

**Software:** Yu Liu.

**Validation:** Hongzhen Wu.

**Writing – original draft:** Hongzhen Wu, Xinqing Jiang, Xinhua Wei, Weifeng Liu, Yuan Guo.

**Writing – review and editing:** Hongzhen Wu, Yingying Liang, Xinqing Jiang, Xinhua Wei, Yu Liu, Weifeng Liu, Yuan Guo, Wenjie Tang.
